# Socio‐ecological benefits of fine‐flavor cacao in its center of origin

**DOI:** 10.1111/conl.12936

**Published:** 2022-12-28

**Authors:** Teja Tscharntke, Carolina Ocampo‐Ariza, Justine Vansynghel, Blanca Ivañez‐Ballesteros, Pablo Aycart, Lily Rodriguez, Marleni Ramirez, Ingolf Steffan‐Dewenter, Bea Maas, Evert Thomas

**Affiliations:** ^1^ Department of Agroecology University of Göttingen Göttingen Germany; ^2^ Alliance of Bioversity International and CIAT, Lima office Lima Peru; ^3^ Department of Animal Ecology and Tropical Biology Biocenter, University of Würzburg Würzburg Germany; ^4^ Department of Botany and Biodiversity Research University of Vienna Vienna Austria; ^5^ Centro de conservación, investigación y manejo de áreas naturales, CIMA Lima Peru

**Keywords:** agroecosystem resilience, biodiversity‐friendly land use, biological pest control, crop domestication, crop pollination, cultural importance, ecological and economic needs, ecosystem services, genotype selection and conservation, tropical agroforestry

## Abstract

In the tropics, combining food security with biodiversity conservation remains a major challenge. Tropical agroforestry systems are among the most biodiversity‐friendly and productive land‐use systems, and 70% of cocoa is grown by >6 million smallholder farmers living on <2$ per day. In cacao's main centre of diversification, the western Amazon region, interest is growing to achieve premium prices with the conversion of high‐yielding, but mostly bulk‐quality cacao to native fine‐flavor cacao varieties, culturally important since pre‐Columbian times. Conversion to native cacao can be expected to favor adaptation to regional climate and growth conditions, and to enhance native biodiversity and ecosystem services such as biological pest control and pollination, but possibly also imply susceptibility to diseases. Experience from successful conversion of non‐native cacao plantations to fine‐flavor cacao agroforestry with rejuvenation by grafting and under medium‐canopy cover levels (30%–40%) can ensure a smooth transition with only minor temporary productivity gaps. This includes ongoing selection programs of high yielding and disease resistant native fine‐flavor cacao genotypes and organizing in cooperatives to buffer the high market volatility. In conclusion, the recent interest on converting bulk cacao to a diversity of native fine‐flavor varieties in countries like Peru is a challenge, but offers promising socio‐ecological perspectives.

## 1 INTRODUCTION

Ongoing large‐scale land‐use intensification and expansion cause dramatic biodiversity losses, while poverty and hunger are still major global issues (Terlau et al., 2019). Large‐scale farming is often perceived as pivotal for feeding the world, but in reality, small‐scale farmers are the backbone of global food security and achieve more socio‐ecological benefits than large‐scale farmers, including higher yields and higher biodiversity (Ricciardi et al., 2021; Terlau et al., 2019; Tscharntke et al., 2012). In the tropics, combining food security with biodiversity conservation remains a major challenge, and many land‐use types are mainly devoted to only maximizing productivity (Grass et al., 2020). Tropical agroforestry systems, such as cacao (*Theobroma cacao*) agroforestry, are more biodiverse and sustainable than any other land‐use system (Tscharntke et al., 2011), in particular when grown with shade tree cover of ca. 40% and in landscapes with forest remnants (Clough et al., 2011; Niether et al., 2020). Seventy percent of cacao is grown by >6 million smallholder farmers in the lowland tropics of Latin America, West Africa and Indonesia, living on <2$ per day and relying on cacao for 60%–90% of their income (Díaz‐Valderrama et al., 2020; Voora et al., 2019). The socio‐ecological standards of cacao production are highly variable (Maas et al., 2020), mainly due to increasing global demand and price pressure favoring full sun monocultures, often in combination with questionable labor conditions and narrow profit margins. High demand for tropical cash crops comes often at the cost of quality, and there is growing concern of decreasing quality standards for international markets (Saltini et al., 2013). The growing interest in single‐origin fine‐flavor cacao (see the Cocoa of Excellence Programme, www.cocoaofexcellence.org) appears to be a great opportunity for smallholders in South America where a plethora of native fine‐flavor varieties occurs to specialize in markets with premium prices, as has been achieved for coffee (Hernandez‐Aguilera et al., 2018). Latin America is the dominant global source (ca. 80%) of fine‐flavor cacao, but only 5% of the global market is made up by fine‐flavor cacao (Ceccarelli et al., 2022). The recently developed Peruvian national plan for the development of the value chain of cacao and chocolate toward 2030 by the Ministry of Agriculture in Peru includes promotion of native cacao varieties, as Peru is recognized for its high offer of fine flavor cacao of sustainable origin (Ministerium de Desarrollo Agrarian y Rigor, 2021). The vision of the plan includes that cacao producers cultivate high‐quality cocoa in agroforestry systems and in diverse landscapes generating ecosystem services and that they receive sufficient revenue for a high quality of life.

**FIGURE 1:**
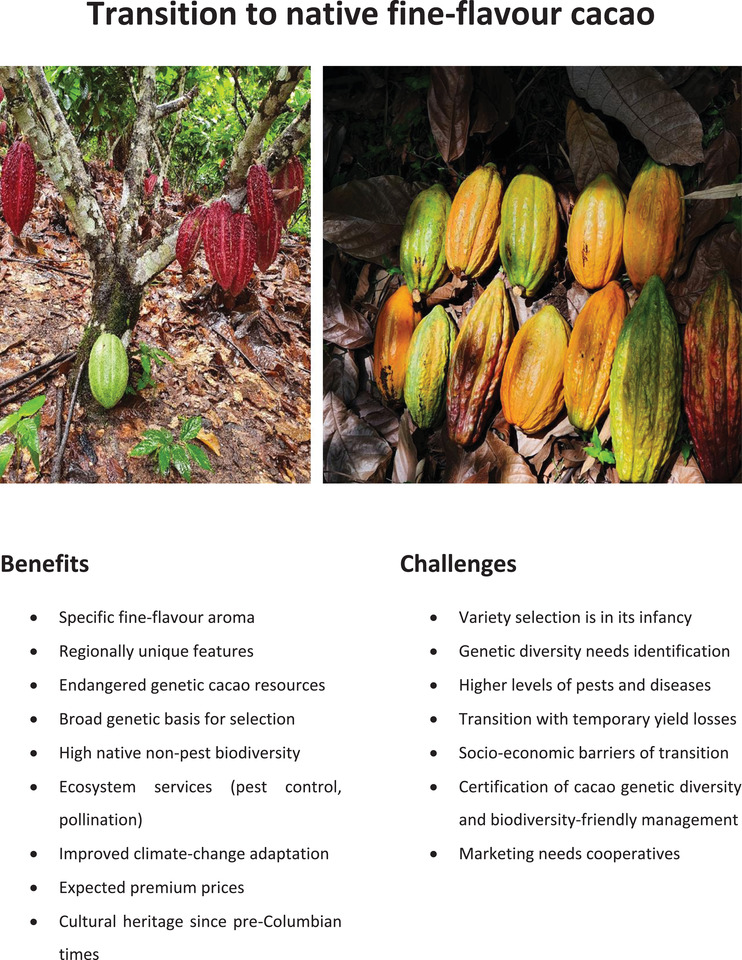
Benefits and challenges of the transition to native fine‐flavor cacao. All topics are discussed in the text, including references. Photos of cacao fruits from Peru: (left) Cacao tree grafted with the non‐native, high‐yielding clone CCN51 America (dark red), while on the rootstock, below the point of grafting with scions, a native cacao fruit (green) developed. (Right) A diversity of cacao fruits from different native varieties (Copyright: Teja Tscharntke)

So far, most cacao is produced outside its center of origin, and South America produces only 12.8% of the cacao traded on the world market (Díaz‐Valderrama et al., 2020). However, the genetic potential of wild cacao populations and managed native cacao varieties in the countries of origin is currently threatened by the introduction of foreign germplasm (Solorzano et al., 2012), the destruction of forest sites with wild cacao populations, and deficient efforts to identify and protect the remaining genetic diversity (Chumacero de Schawe et al., 2013). Local crop genetic resources and landraces are often endangered (Brush, 1995; Ceccarelli et al., 2022; Ficiciyan et al., 2018), although the high socio‐ecological potential of neglected and underutilized crop varieties and species is well acknowledged (Hunter et al., 2019). Here, we characterize the geographic distribution of native fine‐flavor cacao varieties as well as their benefits and constraints. We also address the challenge of selecting and implementing the most promising varieties and end with a discussion of the ecological, social, and economic opportunities of promoting native fine‐flavor cacao (Figure 1). We aim at providing broad socio‐ecological evidence in favor of a transformation of bulk to fine‐flavor cacao in the cacao's countries of origin, supporting the interest of smallholder farmers, cooperatives, and governments alike. This goal is in line with recent efforts to protect and develop crop genetic diversity for global Sustainable Development Goals (SDG), including enhancement of native biodiversity and associated ecosystem services (Dulloo et al., 2021).

## 2 NATIVE FINE‐FLAVOR CACAO: BENEFITS AND CONSTRAINTS

**FIGURE 2:**
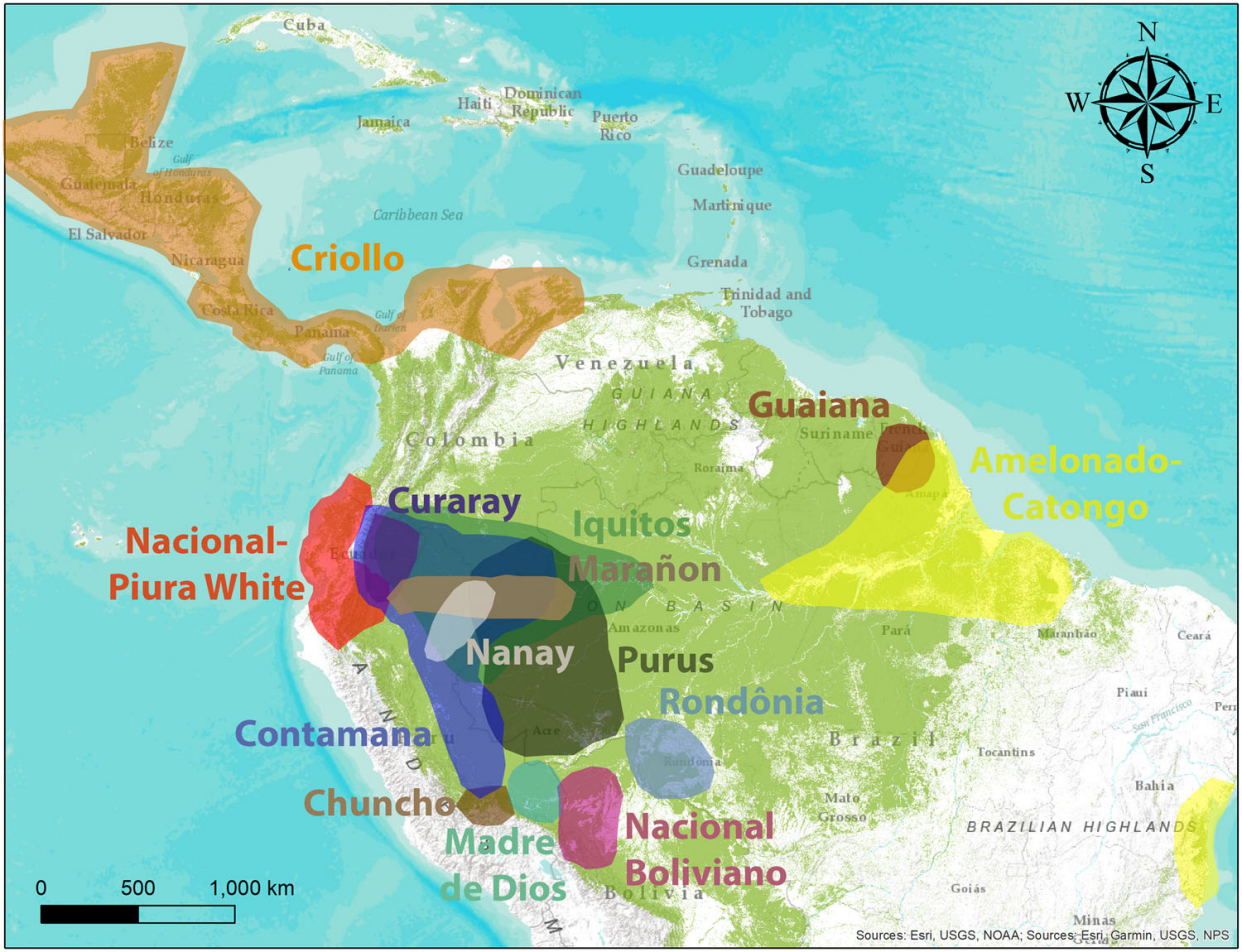
The distribution of cacao genetic groups in their center of origin based on Motamayor et al. (2008), Thomas et al. (2012), Zhang et al. (2012), and Arevalo‐Gardini et al. (2019). Because of limited knowledge of the genetic groups, this is an underrepresentation of the true diversity in the cacao germplasm.

The cacao tree originated in the understorey of the Amazon rainforest, where wild cacao stands can still be found, but the geographical distribution of managed cacao has expanded pantropically (Zhang & Motilal, 2016). Cacao diversified genetically in western Amazonia (Thomas et al., 2012) and has been first domesticated in the region covering southern Ecuadorian and northern Peruvian Amazon before it was introduced to Central America (Zarrillo et al., 2018). Hence, Peru, Bolivia, Venezuela, Ecuador, Colombia, and Brazil are home countries of cacao (Ceccarelli et al., 2022; Saltini et al., 2013). Formerly, cacao has been classified into three major groups: Criollo, which has been cultivated in pre‐Columbian Mesoamerica, where it was introduced with few individuals from South America; Forastero, which refers to all other types of cacao; and Trinitario, which means hybrids between Criollo and Forastero (Díaz‐Valderrama et al., 2020). In recent years, this outdated classification has been replaced by a more detailed one, based on the results of molecular analyses of cacao germplasm with microsatellites, which have identified 11 main genetic groups: Criollo, Marañon, Curaray, Iquitos, Nanay, Contamana, Madre de Dios, Amelonado‐Catango, Purus, Rondônia, Guiana, Nacional‐Piura White, and Nacional Boliviano (Arevalo‐Gardini et al., 2019; Motamayor et al., 2008; Thomas et al., 2012; Zhang et al., 2012) (Figure 2). However, this classification still does not adequately describe the existing diversity in cacao. For example in Peru, multiple native fine‐flavor cacao varieties have been recognized at the national level, of which the best known are Chuncho cacao (pertaining to the Contamana genetic group; mostly in the southern Andean foothills of Cusco, e.g., www.perupuro.de, https://originalbeans.de), Piura white cacao (Nacional genetic group; in the north‐western coastal plains, e.g., https://cocoarunners.com/grower/norandino/), and Amazonas cacao (of mixed genetic origin, but strong Nacional Piura‐White background; Bustamante et al., 2022). Different cacao varieties exhibit different flavor profiles (Saltini et al., 2013) (www.finechocolateindustry.org/about_fine_chocolate.php; www.icco.org/fine‐or‐flavor‐cocoa/).

Maintaining the genetic diversity of cacao in its center of origin is of global importance, first and foremost for breeding purposes and the future selection of new, improved crop varieties (Ficiciyan et al., 2018). Although 87.2% of all cacao beans are produced outside the countries of origin (Africa: 70.2%, Asia 13%, South America 12.8%, Central America 2.9%), native varieties offer the opportunity for high‐quality products with a market transformation toward a unique selling feature (Díaz‐Valderrama et al., 2020). In the 19th century, ca. 95% of all cacao was considered fine quality, whereas nowadays 95% is low‐quality bulk cacao and just 5% fine‐flavour (Díaz‐Valderrama et al., 2020). Specialization on regionally unique varieties with particular tastes may allow high market benefits as is known from many other commodities (Waehning & Filieri, 2022).

Native varieties of cacao are highly valued also because of their cultural (*Theobroma* comes from the Greek “Food of Gods”), medical, and spiritual importance. Cacao, or chocolate, has been advocated for >100 medicinal uses, including gaining weight, stimulating the nervous system, and improving digestion (Dillinger et al., 2000). Cacao played a role already in pre‐Columbian South America with wild cacao used for the pulp, eaten directly or used for fermented beverages. Archaeological evidence revealed early interactions between coastal and Amazonian cacao growers (Zarrillo et al., 2018). During the 15th century, the Incas subordinated the “Chachapoyas” to get access to major tropical food resources, including cacao (Díaz‐Valderrama et al., 2020). This was likely the native “Cacao Amazonas Peru” variety, which has obtained the status of protected denomination of origin in Peru (Bustamante et al., 2022; Ministerium de Desarrollo Agrarian y Rigor, 2021). Hence, preference for this variety as well as for Peruvian Chuncho and Nacional Piura‐white cacao is also influenced by farmers’ interest in keeping the endangered cultural heritage of a unique cacao diversity of regional and national importance.

While crop domestication involves selection for beneficial traits, including sensorial traits, yield, harvest time, resource use as well as pest and disease resistance (Macfadyen & Bohan, 2010), wild or little‐domesticated native crop varieties may be better adapted to the use of local resources and to their abiotic and biotic environment. This includes higher mitigation abilities to climate change (Ceccarelli et al., 2021). Native crops typically allow also more species‐rich associations of crop plants with non‐pest organisms such as insects and other arthropods (Keane & Crawley, 2002). Smallholders tend to implement diversified cropping systems and cultivation methods, which is greatly enhancing biodiversity, and to manage their fields less intensively, which allows survival of more plant and animal species (Rakotomalala et al., 2023; Ricciardi et al., 2021). This includes keeping natural shade trees with canopy cover at medium levels (ca. 30%–40%), supporting higher biodiversity such as birds (Clough et al., 2011), herbaceous plant communities and litter for higher ground‐living biodiversity (Clough et al., 2010; Wanger et al., 2009, 2011). Shaded cacao agroforestry also facilitates native non‐pest insects, including predatory insects such as ants, causing up to 34% yield gains (Wielgoss et al., 2014), and insectivorous birds and bats, which are also major biocontrol agents increasing yield by 30%–100% (Maas et al., 2013, 2016; Vansynghel, Ocampo‐Ariza, Maas, Martin, Thomas, Hanf‐Dressler, Schumacher, Ulloque‐Samatelo, Tscharntke, et al., 2022). Pollinator populations are also a major leverage point to enhance cacao productivity, as very limited pollination success restricts yields (Vansynghel, Ocampo‐Ariza, Maas, Martin, Thomas, Hanf‐Dressler, Schumacher, Ulloque‐Samatelo, Tschantke, et al., 2022; Chumacero de Schawe et al., 2013), shown by hand pollination, increasing yields by up to 200% (Groeneveld et al., 2010; Toledo‐Hernández et al., 2020). Diversifying production systems with several native cacao varieties (Bennett, 2003) should increase pollination success due to the portfolio effect of higher pollen diversity (Paschke et al., 2002). In contrast, domestication is known to often disrupt food webs, reduce resistance, and change volatile blends, negatively affecting prey‐finding by natural enemies (Macfadyen & Bohan, 2010). Further, regarding spatial scale, enhancing biodiversity does not only need biodiversity‐friendly local management, but also surrounding landscapes with forest remnants (Clough et al., 2010; Tscharntke et al., 2015; Klein et al., 2002).

Currently, 56% of the cacao area in Peru is cultivated with the non‐native clone CCN‐51, which has been introduced since the 1970s as a highly productive result of yield‐focused selection and breeding. Still, 44% of the cacao area is planted with native cacao; similar figures are known from Ecuador, as CCN‐51 currently covers 60% of the cacao area, leaving only 40% for Ecuador's Cacao Nacional (Ceccarelli et al., 2022). In Peru, 54% of farmers produce propagation material on their farms, and only 15% obtain it from the 39 nurseries in the country, whereas in Ecuador, most farmers rely on the country‐wide 554 nurseries (Ceccarelli et al., 2022). Challenges of using native varieties include the risk of lower and more variable productivity, as native varieties have often not yet undergone a strict selection process for high yields (Zhang & Motilal, 2016). Hence, acceptance of native varieties by farmers can be difficult, as their trust in productivity outcomes is limited, in particular due to the uncertainty of long‐term performance of hitherto unknown varieties. For example, pollination and fruit set may not be always enhanced in native varieties (Vansynghel, Ocampo‐Ariza, Maas, Martin, Thomas, Hanf‐Dressler, Schumacher, Ulloque‐Samatelo, Tscharntke, et al., 2022; Chumacero de Schawe et al., 2018), and possible differences in susceptibility or resistance to pests and diseases are also not well explored (Hebbar, 2007). Cacao diseases account for ca. 38% of global losses of cacao harvest (Marelli et al., 2019), as cacao resistance levels to diseases are generally low. Some of the causal agents are globally distributed (e.g., *Phytophtora palmivora*), whereas others have geographically restricted populations (Marelli et al., 2019). Major cacao diseases such as Witches’ Broom (*Moniliophtera perniciosa*, previously *Crinipellis* sp.) and Frosty Pod Rot (*Moniliophtera roreri*), so far limited to parts of Latin America, are still absent in major production regions, and eventual invasion to Africa and Asia may seriously change cacao's global productivity (Díaz‐Valderrama et al., 2020). The devastating effects of these diseases have been, for example, shown by the invasion of Witches’ Broom in Bahia (Brazil), followed by a dramatic decline in cacao productivity up to today (Clough et al., 2009; Tscharntke et al., 2011). Therefore, considering the tolerance and resistance of local varieties to pest and diseases can greatly improve our ability to develop genetically stable varieties with both valuable flavor and high resilience. There is a need to better characterize genetic cacao material and to promote selection and breeding programs for native fine‐flavour cacao varieties, which can be demonstrated to smallholders and cooperatives as example for highly productive farming with resilience toward environmental change.

## 3 SELECTION AND IMPLEMENTATION OF THE BEST NATIVE CACAO VARIETIES

Cacao bean flavor is of major importance for producing and marketing high‐level cacao beans and products such as chocolate (Kongor et al., 2016). The cacao flavor is not only influenced by the genetic makeup of a variety, but also by tree age, soil chemical composition, local climatic conditions, and postharvest treatments such as fermentation and roasting, but the relative contribution of each factor remains unclear (Kongor et al., 2016; Lima et al., 2011). Before smallholders decide for the on‐farm implementation of native fine‐flavour cacao, the most suitable and productive cacao varieties need to be identified and selected. This requires detailed genetic, physical, and sensorial analyses of the native fine‐flavour cacao varieties, a process that still needs to be optimized (Motamayor et al., 2008) and then implemented in a socio‐ecologically responsible way. Conversion of current cacao plantations, dominated by high‐yielding clones and varieties that result from (inter)national breeding programs (e.g., CCN51; Jaimez et al., 2022), toward >30 new native fine‐flavour cacao genotypes or varieties across >10,000 ha has been acknowledged as a major goal of agricultural policy in Peru (Ministerium de Desarrollo Agrarian y Rigor, 2021). One of the first steps includes establishment of more and improved gene banks and working collections (generally, clonal gardens) (Apshara, 2017; Ceccarelli et al., 2022; Sena Gomes et al., 2015). Keeping a diversity of flavor profiles in situ means higher flexibility to respond to changing future interests for adaptations to market or environmental changes. Further, live collections on‐farm, holding high genetic diversity combining relict old trees (i.e., the old genetic basis) and new (grafted) varieties, may advance economic and ecological resilience. Using clonal propagation through grafting instead of growing new plantations from seedlings grown in seed gardens avoids long productivity gaps in favor of quick recovery of cacao productivity (Vaast & Somarriba, 2014; Zavaleta et al., 2022). Combining different genotypes in the rootstock (the mother tree) and the scions, grafted on the pruned mother tree, may be optimized to maximize pollination success and fruit set (Vansynghel, Ocampo‐Ariza, Maas, Martin, Thomas, Hanf‐Dressler, Schumacher, Ulloque‐Samatelo, Tscharntke, et al., 2022). Grafting of old trees can be easily performed with standard grafting practices (Daouda et al., 2018), and field experiments with Piura white cacao (Ocampo‐Ariza et al., 2022) showed that after ca. 2 years yield levels can be 40%–80% higher than in non‐grafted neighboring trees (unpublished data). This successful transformation of bulk cacao to fine‐flavour and native varieties was carried out by farmers in the Piura region, organized in the large cooperative Norandino (with >5400 producers; Maas et al., 2020) and provides a promising example for the whole country.

## 4 BALANCING SOCIO‐ECOLOGICAL NEEDS WITH IMPLEMENTING NATIVE FINE‐FLAVOUR CACAO

Replacing high‐yielding, bulk‐quality cacao by native fine‐flavour varieties in the species´ center of origin appears to be a promising strategy for land‐use development meeting the SDG, although some frame conditions are still unclear (see above). The socio‐ecological importance of maintaining and rescuing genetic diversity in the crop's native range is well recognized (Ceccarelli et al., 2022; Ficiciyan et al., 2018). Further, the unique flavor of geographically restricted cacao varieties (such as single‐origin cacao from Chuncho and Piura white cacao in Peru) may allow for marketing advantages with certified products and premium prices. Challenges include the future agronomic optimization and the so far little developed market with its great price volatility (e.g., from 3422 to 1769$ per ton within two years: 2015–2017) (Voora et al., 2019). Fine‐flavour cacao still lacks major selection programs for high yields and disease resistance, as local diseases are inhibiting expansion of the cacao sector in South America. While fine‐flavour cacao is still a small segment in the global cacao trade, it has been growing faster than the bulk market at rates between 7% and 11% (compared to 2%–3%) per year since 2011 (Vignati & Gómez‐García, 2020). In times of unprecedented global losses of biodiversity, biodiversity‐friendly management of native cacao, including their native and endemic species as well as associated ecosystem services such as pest control, can become a major part of the marketing strategy (Maas et al., 2020; Tscharntke et al., 2015).

The discussion about replacing high‐yielding bulk‐quality cacao varieties by fine‐flavour native varieties is often dominated by agronomic aspects, in particular the limited knowledge of productivity, compatibility between varieties and climate resilience (Lahive et al., 2018), although native fine‐flavour varieties appear to be more climate resilient than bulk cacao (Ceccarelli et al., 2021). Clearly, considerations of smallholders in face of such a long‐term decision are largely determined by social and economic aspects (Toledo‐Hernández et al., 2021). Converting or restructuring small farms with tree crops such as cacao means a long‐term investment and commitment, as productivity may reach its optimal level only after a few years, but the grafted and thereby rejuvenated cacao trees ensure higher income already within few years. In contrast to grafting, rejuvenating cacao plantations from seeds means a much longer yield gap, and higher risk for the outcome. Grafting, but not the replanting with cacao seedlings or saplings, allows for a relatively quick change in farm strategies due to political change, price volatility, or social stress situations. More regional field trials and best practice examples are required to identify the most promising combinations of native varieties and to convince farmers of the benefits of changing their system. Smallholder farmers’ livelihoods depend on their small piece of land, meaning there is little resilience to extreme events or changing environmental and socioeconomic conditions – as far as smallholders are not organized in cooperatives (Maas et al., 2020). Organization in cooperatives as well as eco‐labeling (such as organic and fairtrade, with certification costs shared within the cooperative) may be particularly helpful to overcome insecurities of small‐scale farming (Maas et al., 2020; Silva et al., 2017). Successful and sustainable eco‐labeling for biodiversity needs maintenance or restoration of shade trees and forest remnants in the surrounding landscapes, but may further increase premium prices (Tscharntke et al., 2015; Waldron et al., 2015).

In conclusion, replacing high‐yielding bulk‐quality cacao by fine‐flavour native varieties, grown in cacao agroforests, contributes to the conservation of cacao genetic diversity, associated diversity of non‐pest organisms and their ecosystem services as well as cultural heritage and may allow marketing advantages of unique regional products. Hence, current challenges associated with the conversion toward native varieties need to be tackled because of the high socio‐ecological value of such a transition. Promoting the diversity of cacao varieties in a diversified agroforestry system, as done recently by the Peruvian government and farmers’ cooperatives, has a major potential for a biodiversity‐friendly and socially sustainable development of smallholder farming in the cacao's native range.
